# PReS-FINAL-2171: Consensus: what agent to use when first-line vasodilators fail in Raynaud's phenomenon or digital ulcers secondary to rheumatic diseases in children?

**DOI:** 10.1186/1546-0096-11-S2-P183

**Published:** 2013-12-05

**Authors:** M Katsicas, M Gonzalez, R Russo

**Affiliations:** 1 Hospital de Pediatrìa Prof Dr JP Garrahan, Buenos Aires, Argentina

## Introduction

Juvenile Systemic Sclerosis (JSS) is characterized by Raynaud's phenomenon (RP) and digital ulcers (DU). Conventional therapy includes calcium channel blockers (CCB) A growing number of vasodilators is available for treatment of refractory patients but there is no clear evidence of the best option. To aid clinical decision-making, a consensus of expert was undertaken.

## Objectives

To identify the best therapeutic options and define the sequence of 2^nd ^line vasodilators for RP and DU.

## Methods

Steps in the process of consensus were: a) Identification of expert panel (EP) members, b) Identification of 2^nd ^line vasodilators c) identification of outcome measures to define RP and DU improvement, d) systematic literature review; e) summary report of the latest scientific evidence f) expert consensus meeting; g) rating of the strength of evidence. RAND/UCLA appropriateness method was used for rating the medical decision: items were rated on a 9-point scale on each drug option. There were two scoring rounds: first: anonymous and independent rating of the appropriateness of vasodilators based on scientific evidence and best clinical judgment. Differences in scoring were discussed at a face-to-face meeting, followed by a second rating round. Consensus was reached on appropriate/inappropriateness.

## Results

The EP included 10 physicians from a tertiary center who are involved in the care of patients with JSS: 3 pediatric rheumatologists, 2 dermatologists, 1 pediatrician, 1 gastroenterologist, 1 nephrologist, 1 nutricionist, 1 pharmacologist, and a moderator. The EP identified 4 drugs for analysis: bosentan, iloprost, sildenafil, and trepostinil. Outcome measures were selected according to the literature references and EP judgment. RP improvement definition: ≥ 30% improvement according to the physician (in a visual analogue scale, VAS) and ≥ 30% improvement in at least 2 patient-related domains (pain or function). Patient domains were: a) number of episodes, b) pain in a VAS, c) function (impaired activity of daily living, VAS), d) RP episodes average duration (in minutes). DU improvement definition: a favorable change in all physician- and patient-related domains: patient's domains: a) pain (VAS) b) function (VAS); physician's domains: a) ulcer activity (VAS) b) horizontal and transverse DU diameter (in mm). Systematic literature review was performed independently by 5 EP members and guided by the moderator. All articles in English were eligible. Data bases included pubmed and Cochrane. The search strategy included all relevant terms: bosentan, iloprost, sildenafil, trepostinil, RP, DU, combined in different sets of keywords. The summary report of the scientific evidence included 25 articles. Ranking of papers according to the strength of evidence showed: 1a (1 paper), 1b (7), 2b (2), 3b (2), 4(8), 5(5). After second scoring round: 1^st ^appropriate indication Iloprost; 2^nd ^bosentan, 3^rd ^sildenafil; 4^th ^trepostinil.

## Conclusion

The EP reached a consensus on vasodilator drugs, providing direction for common dilemmas in the pharmacologic treatment of RP and DU in refractory patients.

## Disclosure of interest

None declared.

